# Management guidelines for pregnant women living with epilepsy: An integrative literature review

**DOI:** 10.4102/hsag.v29i0.2772

**Published:** 2024-12-06

**Authors:** Sumeshni Birbal, Frasia Oosthuizen

**Affiliations:** 1 Discipline of Pharmaceutical Sciences, College of Health Sciences, University of KwaZulu-Natal, Durban, South Africa

**Keywords:** epilepsy, pregnancy, anti-epileptic drugs, major congenital malformations, therapeutic drug monitoring

## Abstract

**Background:**

Epilepsy affects more than 50 million people worldwide, with a significant number of safety-related concerns arising in pregnant women. Precise management needs to include a vast array of stepwise protocols; however, no studies have been conducted to compare current South African guidelines to international norms.

**Aim:**

The aim of this integrative literature review was to identify discrepancies, if any, in the current protocols for treating pregnant women with epilepsy within the public sector of South Africa when measured against global standards.

**Method:**

An integrative literature review was conducted using the Whittemore and Knafl method. The following databases and search engines were used: PubMed, EBSCOhost, Science Direct, Medscape, African Journals Online, Cochrane Library, Google scholar, Google and MedlinePlus. The study included qualitative literature from all levels of evidence from 2006 to 2023. The data were evaluated using the PRISMA flow chart and analysed using the Cooper method. On completion of critical appraisal, 22 articles met the inclusion criteria and were analysed thematically.

**Results:**

The review identified several areas that can be enhanced to improve patient management and outcomes such as therapeutic drug monitoring and targeted dosing, folic acid supplementation, the use of antepartum vitamin K, high-resolution ultrasounds, elimination of sodium valproate in women of childbearing potential, monotherapy and endorsing frequent obstetrician visits.

**Conclusion:**

The study validated many differences between guidelines used in South Africa and internationally. Some of the prominent findings included areas focused on patient medication, screening, physician visits and pharmacovigilance.

**Contribution:**

The findings can be used to enhance and provide evidence-based recommendations in the areas that are not well developed within the current guidelines.

## Introduction

The clinical management of pregnant women with epilepsy (WWE) remains a global health challenge (Tamar 2020). The World Health Organization (WHO) defines epilepsy as a chronic non-communicable disease of the brain, characterised by recurrent seizures (WHO 2023). Epilepsy accounts for a significant proportion of the world’s disease burden, affecting more than 50 million people worldwide (Liu et al. 2023). The estimated proportion of the general population with active epilepsy at a given time is between 4 and 10 per 1000 people (Owolabi et al. 2020). According to the WHO, an estimated 2.4 million people globally are diagnosed with epilepsy each year (WHO 2023). In high-income countries (e.g. United Kingdom [UK], United States [US]) annual new cases are between 30 and 50 per 100 000 people in the general population (Owolabi et al. 2020). In low- and middle-income countries (South Africa [SA] included), this figure can be up to two times higher (Owolabi et al. 2020).

Anti-epileptic drugs (AEDs) are an essential component in the clinical management of epilepsy and control seizures in approximately 7 out of 10 people (WHO 2023). The alteration of brain electrochemical activity helps control seizures, thereby preventing untoward neurological sequelae (Liu et al. 2023). Anti-epileptic drugs cannot cure epilepsy but provide substantial benefits that lead to an improved quality of life (Liu et al. 2023). However, in addition to the medicine safety issues that apply whenever AEDs are taken by patients, additional concerns may arise in pregnant women (Tamar 2020). Foetal exposure to some, but not all, AEDs may be associated with the development of various malformations in the foetus and, at least in the case of sodium valproate, with postnatal neurodevelopmental delay, lower IQ values and sometimes autism-spectrum disorders (Gedezelman & Meador 2012; South Australian Perinatal Guideline 2018). Severe abnormalities that will need treatment after birth or lead to long-term health problems include malformations of the spinal cord and heart, as well as cleft lip and palate (South Australian Perinatal Guideline 2018; Watila et al. 2018). Other minor birth defects, which primarily affect the baby’s appearance (dysmorphic features), include facial abnormalities, such as eyes set too wide apart, a short upper lip, small fingers and toes with rudimentary nails (Kuluga et al. 2018; Watila et al. 2018). The abnormalities range from comparatively trivial deformities of the skin to potential surgically remediable malformations to devastating and uncorrectable abnormalities – some being incompatible with continuing extrauterine existence (Kuluga et al. 2018; Watila et al. 2018).

The impact of maternal AEDs on cognitive and behavioural development of the foetus is of concern (Borgelt, Hart & Bainbridge 2019; Patel & Pannell 2016). Research has established that sodium valproate is teratogenic and, therefore, should be avoided in women of childbearing age if possible (Eadie 2020).

In the UK, the Medicines and Health Products Regulatory Agency restricted the use of sodium valproate in pregnant women in April 2018 (Tamar 2020). In December 2018, the South African Health Products Regulatory Authority (SAHPRA) issued a statement that advised that women of childbearing age should not be started on sodium valproate treatment unless no other effective treatment is available, and those already taking the drug should be made aware of the risks and take precautions to avoid falling pregnant (Tamar 2020).

According to SAHPRA, an estimated one in 10 babies exposed to sodium valproate in utero are likely to develop serious birth defects, such as spina bifida, facial and skull deformities, and heart, kidney and limb defects (Tamar 2020). Up to 40% of babies whose mothers took sodium valproate during pregnancy are at risk of developmental problems such as delayed speech and walking (Borgelt et al. 2019; Watila et al. 2018). They are also at a greater risk of having autism spectrum disorders and attention-deficit hyperactivity disorders (Tamar 2020). Thus, appropriate management of pregnant WWE is imperative and needs to include a vast array of stepwise protocols, such as patient education, pre-natal supplementation, frequent therapeutic drug monitoring, as well as the prescribing of safer drugs (Harden et al. 2009; Pennell et al. 2022).

Current South African guidelines for the management of epilepsy include guidelines for the general population and a step-wise medicine treatment, specific to the management of pregnant WWE (NDoH 2020).

### Aim

The aim of this integrative literature review was to identify discrepancies, if any, in the current protocols for treating pregnant WWE within the public sector of South Africa when measured against global standards.

### Objectives

1. To conduct a review of the international management guidelines for pregnant WWE
2. To conduct a review of the guidelines currently in place for use within the public healthcare sector of KwaZulu-Natal, South Africa
3. To highlight possible shortcomings within the current public sector guidelines

## Methods

The review was conducted in accordance with the Whittemore and Knafl’s methodology for integrative literature reviews and included the following five stages: (1) identification of the research problem, (2) search of the literature, (3) evaluation of the data, (4) analysis of the data and (5) presentation of the results (Whittemore & Knafl 2005).

### Stage one: Identification of the research problem

A focused review question was formulated using the PICO acronym as follows:

P – (population or participants) = Pregnant epileptic patients

I – (Intervention) = Guidelines to manage pregnant epileptic patients

C – (Comparison) = Guidelines used to manage pregnant epileptic patients, internationally

O – (Outcome) = Improve the guidelines being used to manage pregnant epileptic patients

The review question that was formulated to search the relevant literature was the following:

1. *What guidelines are currently available to manage pregnant epileptic patients within the public sector in KwaZulu-Natal?*
2. *How do these guidelines compare to guidelines being used internationally?*

### Stage two: Search of the literature

The appraisal was conducted to review the management protocols and prescribing guidelines for pregnant WWE in SA and other countries. This was performed to determine whether guidelines for pregnant WWE existed in SA and to draw comparisons with international guidelines. The following areas were analysed:

- Seizure frequency during pregnancy and the risk associated in pregnant WWE
- AED-induced harm to the foetus
- Pre-natal supplementation and favourable AEDs that can be used during pregnancy
- High-risk AEDs that should be avoided during pregnancy
- Monotherapy of AEDs versus polytherapy
- Therapeutic drug monitoring and pre-natal screening
- High-resolution ultrasounds
- Recommended neurologist and/or physician visits

The following databases and search engines were used: PubMed, EBSCOhost, Science Direct, Medscape, African Journals Online, Cochrane Library, Google scholar, Google, MedlinePlus.

The following search terms were used:

- ‘Management’ of pregnant WWE AND ‘South Africa’
- Epilepsy and pregnancy ‘guidelines’ AND ‘South Africa’
- Guidelines for the management of pregnant epileptic patients
- Step-wise management for pregnant epileptic patients

#### Inclusion criteria

The inclusion criteria for the literature review comprised of literature from all levels of evidence from 2006 to 2023, interconnected to guidelines pertaining to pregnant WWE. The following criteria were applied: (1) Studies published in English, (2) Studies that published results from guidelines, (3) Original studies and guidelines, (4) Only studies with reliable conclusions and intervention methods.

#### Exclusion criteria

All studies that did not pertain to the management of pregnant WWE were excluded.

#### Quality evaluation

**FIGURE 1:**
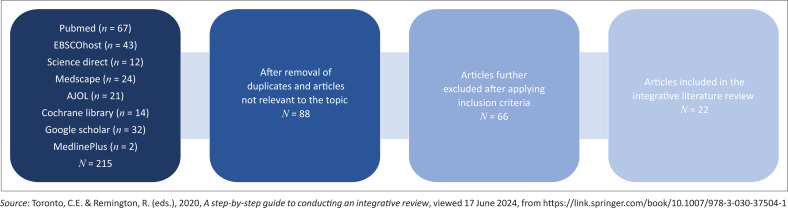
Flowchart summary of the integrative literature review process.

A flowchart of the integrative literature review process is presented in Figure 1.

### Stage three: Evaluation of the data

**FIGURE 2:**
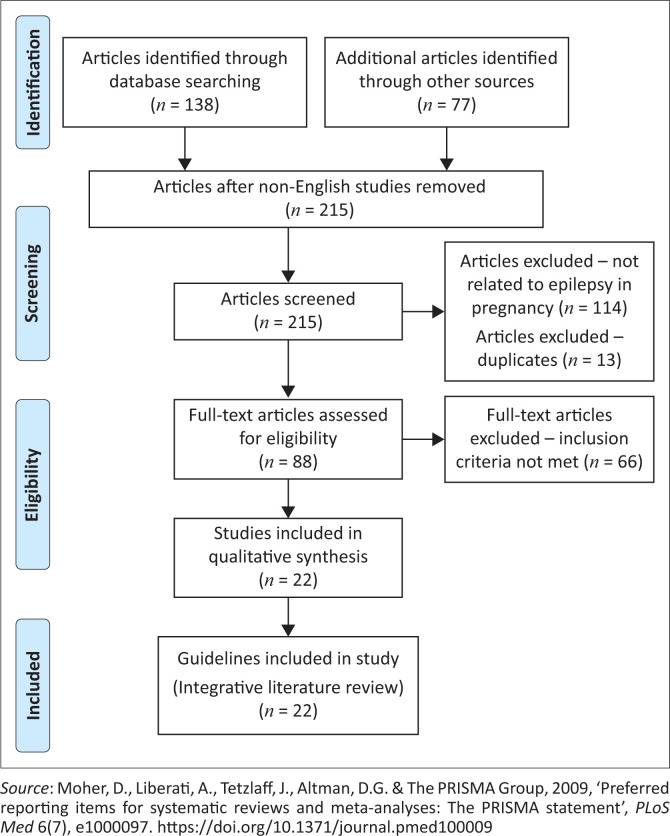
Preferred Reporting Items for Systematic Reviews and Meta-Analyses (PRISMA) flowchart of the search and selection process.

The procedures used in the evaluation process are represented by the Preferred Reporting Items for Systematic Reviews and Meta-Analysis (PRISMA) flowchart (Figure 2). Of the 215 articles that were screened, 127 articles were excluded as they were not pertaining to the management of pregnant WWE (*n* = 114) or they were duplicates (*n* = 13). Following further screening of the guidelines, a total of 88 full-text articles were screened, of which 66 articles did not meet the inclusion criteria, leaving 22 articles for analysis.

### Stage four: Analysis of the data

A thematic analysis of the data was carried out based on the Cooper method (Cooper et al. 2012). This was used to help synthesise the extracted guidelines related to the major recommendations regarding the management of pregnant WWE. The themes identified within the guidelines used for the analysis were grouped and coded according to categories and thereafter compared under themes and sub-themes.

### Stage five: Presentation of the results

The results were grouped and presented in a narrative summary and were supported by the use of tables.

### Ethical considerations

An application for full ethical approval was made to the Biomedical Research Ethics Committee of the University of KwaZulu-Natal, and ethics consent was received on 12 January 2024. The ethics approval number is BREC/00004455/2022.

## Review findings

The themes: (1) Risks, AED and seizure-induced harm during pregnancy, (2) Supplementation and medicine recommendations and (3) Monitoring during pregnancy have been grouped and further divided into sub-themes as displayed in Table 1, Table 2, Table 3 and Table 4.

**TABLE 1:** Department of Health – South Africa guidelines.

Parameter	Component
**Counselling**	
Drug-drug interactions between hormonal contraceptives and AEDs	Progestin subdermal implants and oral contraceptives
Seizure-induced harm during pregnancy	Not mentioned in the guideline
Seizure frequency during pregnancy	Not mentioned in the guideline
Risk associated in pregnant WWE	Not mentioned in the guideline
AED-induced harm to the foetus	Birth defects and persistent development disorders
**Supplementation and medicine recommendations**	
Recommended pre-natal supplementation	Folic acid 5 mg daily
Recommended AEDs during pregnancy	Not mentioned in the guideline
AEDs to be avoided during pregnancy	Sodium valproate
Monotherapy/polytherapy	Monotherapy
**Monitoring**	
Therapeutic drug monitoring	Not recommended, except in certain circumstances (e.g. to confirm drug toxicity). Pregnancy alters AED levels and therefore these need to be adjusted accordingly.
High-resolution ultrasounds	CT scan at the commencement of treatment
OB/GYN visits	Not mentioned in the guideline

*Source:* National Department of Health, South Africa: Essential Drugs Programme. Primary Healthcare Standard Treatment Guideline and Essential Medicine List. 7th ed. South African National Department of Health; 2020

AED, anti-epileptic drug; WWE, women with epilepsy; CT, computed tomography; OB/GYN, obstetrics/gynaecology.

**TABLE 2:** Risks, anti-epileptic drug and seizure-induced harm during pregnancy (international guidelines).

Guideline	Seizure-induced harm during pregnancy	Seizure frequency during pregnancy	Risk associated in pregnant WWE	AED-induced harm to the foetus
**American Academy of Neurology and American Epilepsy Society (AAN & AES)** Harden, C.L., Pennell, P.B., Koppel, B.S., Hovinga, C.A., Gidal, B., Meador, K.J. et al., 2009, ‘Practice parameter update: management issues for women with epilepsy – Focus on pregnancy (an evidence-based review): vitamin K, folic acid, blood levels, and breastfeeding: report of the Quality Standards Subcommittee and Therapeutics and Technology Assessment Subcommittee of the American Academy of Neurology and American Epilepsy Society’, *Neurology* 73(2), 142–149. https://doi.org/10.1212/WNL.0b013e3181a6b325	Status epilepticus – possible in 0% – 1.8% of WWE	Might occur in 14% – 32% of WWE	There is probably a 1.5-fold increased risk of premature contractions, labour and delivery. Neonates have increased risk of low birth weight.	Higher risk of major congenital malformations
**Epilepsy therapy development project – Epilepsy Foundation of America (ETDP-EFA)** Tomson, T., Battino, D., French, J., Harden, C., Holmes, L., Morrow, J. et al., 2007, ‘Antiepileptic drug exposure and major congenital malformations: The role of pregnancy registries’, *Epilepsy & Behavior* 11(3), 277–282. https://doi.org/10.1016/j.yebeh.2007.08.015	Can particularly increase the risk of brain or other injuries in the foetus	Occurs in one-fourth to one-third of WWE	Increased risk of vaginal bleeding, hypertension, preeclampsia, antepartum haemorrhage, and caesarean delivery.	Possible life-threatening risks exists when AEDs not recommended (e.g. sodium valproate) are taken during pregnancy.
**United Kingdom – National Institute for Health and Care Excellence (UK NICE)** Gonzalez-Viana, E., Sen, A., Bonnon, A. & Cross, J.H., 2022, ‘Epilepsies in children, young people, and adults: Summary of updated NICE guidance’, *BMJ* 378. https://doi.org/10.1136/bmj.o1446	Tonic-clonic seizures – relatively higher risk of harm to the foetus	Not likely to increase	More likely to have complications during pregnancy and labour.	Increased risk in majority of patients observed
**Royal College of Obstetricians and Gynaecologists (RCOG) – Green-top guideline no. 68** No 68, G.T.G., 2016, *Epilepsy in pregnancy*, viewed from https://doctor2017.jumedicine.com/wp-content/uploads/sites/7/2021/07/gtg68_epilepsy.pdf.	Association between tonic-clonic seizures and foetal hypoxia, foetal intracranial haemorrhage and foetal loss. There is consistent evidence of an approximate two-fold increased risk of spontaneous abortion, stillbirth and perinatal loss.	Increase in seizure frequency in 15% – 37% of pregnant women.	Induction of labour, instrumental deliveries and caesarean sections may be more common in WWE, when compared to controls.	Most studies show a 2–3 fold increase in major malformations in children exposed to AEDs in-utero, when compared with the general population.
**South Australian Guideline** Morton, M., Rogers, A., Peat, B. & Hotham, N., 2018, *South Australian Maternal, Neonatal, and Gynaecology Community of Practice,* South Australian Perinatal Practice guideline: Epilepsy and Pregnancy management, ISBN: 9781742430676, viewed from: https://www.sahealth.sa.gov.au/wps/wcm/connect/216779804ee459ccb98cbdd150ce4f37/Epilepsy+and+Pregnancy+Management.	Significant risk exists. Foetal hypoxia.	Possible increased frequency during pregnancy and during birth.	A 2 to 3-fold increase in miscarriage, intrauterine growth restriction.	Major and minor congenital malformations, microcephaly.
**North American Registry** Hernández-Díaz, S., Smith, C.R., Shen, A., Mittendorf, R., Hauser, W.A., Yerby, M. et al., 2012, ‘Comparative safety of antiepileptic drugs during pregnancy’, *Neurology* 78(21), 1692–1699. https://doi.org/10.1212/WNL.0b013e3182574f39	There is a substantial increased risk that has been observed.	Data show reassuring results for seizure control during pregnancy.	Haemorrhagic disease of the newborn. WWE are up to four times more likely to have an induced labour and twice as likely to have a caesarean section.	Significant risk of major congenital malformations exists and lower IQ scores in the newborn.
**Australian Pregnancy Registry** Eadie, M.J., 2020, ‘Antiepileptic drug safety in pregnancy: possible dangers for the pregnant woman and her foetus’, *Evaluation* 15, 13. https://doi.org/10.1007/s40120-021-00252-5	Prolonged seizures may result in foetal hypoxia.	Frequency generally not increased with pregnancy.	Risk of miscarriage will be increased.	Exposure to AEDs during pregnancy is associated with adverse development (gross motor skills, sentence skills, autistic traits) at 18 and 36 months of age.
**UK Epilepsy and Pregnancy Register** Morrow, J.I., Russell, A., Guthrie, E., Parsons, L., Robertson, I., Waddell, R. et al., 2006, ‘Malformation risks of antiepileptic drugs in pregnancy: A prospective study from the UK Epilepsy and Pregnancy Register’, *Journal of Neurology, Neurosurgery & Psychiatry* 77(2), 193–198. https://doi.org/10.1136/jnnp.2005.074203	Increases the risk of sudden unexpected death in epilepsy (SUDEP)	Increased risk of tonic-clonic seizures. Every 2–4 per 100 women will experience a seizure during labour or delivery.	Risk of miscarriage, increased seizure frequency, which could result in injuries or harm to the foetus.	Significant risk of congenital malformations exists.
**International Registry of Anti-epileptic Drugs and Pregnancy (EURAP)** EURAP Study Group, 2006, ‘Seizure control and treatment in pregnancy: Observations from the EURAP epilepsy pregnancy registry’, *Neurology* 66(3), 354–360. https://doi.org/10.1212/01.wnl.0000195888.51845.80	Not mentioned	Seizure frequency could be increased. This is patient dependent.	Not mentioned	Major congenital malformations, minor abnormalities, psychomotor and mental development and behaviour affected.

AED, anti-epileptic drug; WWE, women with epilepsy.

**TABLE 3:** Supplementation and medicine recommendations (international guidelines).

Guideline	Pre-natal supplementation (Folic acid)	Pre-natal supplementation (Vitamin K)	Favourable AEDs for use during pregnancy	AEDs that should be avoided during pregnancy	Monotherapy/ Polytherapy
**American Academy of Neurology and American Epilepsy Society (AAN & AES)** Harden, C.L., Pennell, P.B., Koppel, B.S., Hovinga, C.A., Gidal, B., Meador, K.J. et al., 2009, ‘Practice parameter update: management issues for women with epilepsy – Focus on pregnancy (an evidence-based review): Vitamin K, folic acid, blood levels, and breastfeeding: report of the Quality Standards Subcommittee and Therapeutics and Technology Assessment Subcommittee of the American Academy of Neurology and American Epilepsy Society’, *Neurology* 73(2), 142–149. https://doi.org/10.1212/WNL.0b013e3181a6b325	Folic acid 400 mcg/day – prior to conception and during pregnancy may be considered to prevent major congenital malformations.	There is insufficient evidence to support vitamin K supplementation.	Not enough evidence to suggest the use of newer AEDs	Sodium valproate, phenytoin, carbamazepine, phenobarbitone	Avoid polytherapy
**Epilepsy therapy development project – Epilepsy Foundation of America (ETDP-EFA)** Tomson, T., Battino, D., French, J., Harden, C., Holmes, L., Morrow, J. et al., 2007, ‘Antiepileptic drug exposure and major congenital malformations: the role of pregnancy registries’, *Epilepsy & Behavior* 11(3), 277–282. https://doi.org/10.1016/j.yebeh.2007.08.015	Before conception and throughout pregnancy. Recommended dose of 400 mcg/day for non-pregnant women, 600 mcg/day for pregnant women and those contemplating pregnancy and 500 mcg/day for lactating women.	Oral dose of 10 mg/day – 20 mg/day vitamin K suggested during the last month of pregnancy, especially if taking enzyme-inducing AEDs.	Lamotrigine has the most data available at this time, with possibly no substantially increased risk of major congenital malformations. Preliminary studies have found a good pregnancy safety profile with levetiracetam, but more data are needed to draw a conclusion.	Sodium valproate, phenytoin, carbamazepine	Monotherapy preferred
**United Kingdom – National Institute for Health and Care Excellence (UK NICE)** Gonzalez-Viana, E., Sen, A., Bonnon, A. & Cross, J.H., 2022, ‘Epilepsies in children, young people, and adults: summary of updated NICE guidance’, *BMJ* 378. https://doi.org/10.1136/bmj.o1446	Recommended before conception at a dose of 5 mg/day	Not recommended	Not mentioned	Not mentioned	Avoid polytherapy
**Royal College of Obstetricians and Gynaecologists (RCOG) – Green-top guideline no. 68** No, G.T.G., 2016, *Epilepsy in pregnancy,* viewed from https://doctor2017.jumedicine.com/wp-content/uploads/sites/7/2021/07/gtg68_epilepsy.pdf.	Recommended dose of 5 mg/day prior to conception and throughout pregnancy	There is insufficient evidence to recommend (1) routine maternal use of oral vitamin K to prevent haemorrhagic disease of the newborn in WWE taking enzyme-inducing AEDs and (2) giving vitamin K to WWE to prevent postpartum haemorrhage. All babies born to WWE taking enzyme-inducing AEDs should be offered 1 mg of intramuscular vitamin K to prevent haemorrhagic disease of the newborn.	Lamotrigine, carbamazepine	Sodium valproate, phenobarbitone, phenytoin	Avoid polytherapy
**North American Registry** Hernández-Díaz, S., Smith, C.R., Shen, A., Mittendorf, R., Hauser, W.A., Yerby, M. et al., 2012, ‘Comparative safety of antiepileptic drugs during pregnancy’, *Neurology* 78(21), 1692–1699. https://doi.org/10.1212/WNL.0b013e3182574f39	Supplement with 200 mcg/day – 500 mcg/day, ideally 4 weeks pre-gestation and post-conception	Not mentioned	Lamotrigine, levetiracetam	Sodium valproate, phenobarbitone, carbamazepine	Avoid polytherapy especially with sodium valproate.
**Australian Pregnancy Registry** Eadie, M.J., 2020, ‘Antiepileptic drug safety in pregnancy: Possible dangers for the pregnant woman and her foetus’, *Evaluation* 15, 13. https://doi.org/10.1007/s40120-021-00252-5	It is crucial to recommend pre-conception folic acid at least 1 month prior and 4 weeks post-conception.	Not recommended	Lamotrigine, levetiracetam	Phenobarbitone, carbamazepine, phenytoin	Avoid polytherapy – because of higher risk of major congenital malformations
**UK Epilepsy and Pregnancy Register** Morrow, J.I., Russell, A., Guthrie, E., Parsons, L., Robertson, I., Waddell, R. et al., 2006, ‘Malformation risks of antiepileptic drugs in pregnancy: A prospective study from the UK Epilepsy and Pregnancy Register’, *Journal of Neurology, Neurosurgery & Psychiatry* 77(2), 193–198. https://doi.org/10.1136/jnnp.2005.074203	Recommended 500 mcg/day at least 3 months prior to conception	Not mentioned	Not mentioned	Sodium valproate	Avoid polytherapy
**International Registry of Anti-epileptic Drugs and Pregnancy (EURAP)** EURAP Study Group, 2006, ‘Seizure control and treatment in pregnancy: Observations from the EURAP epilepsy pregnancy registry**’,** *Neurology* 66(3), 354–360. https://doi.org/10.1212/01.wnl.0000195888.51845.80	Recommended 5 mg/day	Not mentioned	Lamotrigine, levetiracetam	Sodium valproate, carbamazepine, phenobarbitone, phenytoin	Avoid polytherapy

AED, anti-epileptic drug; WWE, women with epilepsy.

**TABLE 4:** Monitoring during pregnancy (international guidelines).

Guideline	Therapeutic drug monitoring	Pre-natal screening and high-resolution ultrasounds	Neurologist or physician visits
**American Academy of Neurology and American Epilepsy Society (AAN & AES)** Harden, C.L., Pennell, P.B., Koppel, B.S., Hovinga, C.A., Gidal, B., Meador, K.J. et al., 2009, ‘Practice parameter update: management issues for WWE – Focus on pregnancy (an evidence-based review): vitamin K, folic acid, blood levels, and breastfeeding: report of the Quality Standards Subcommittee and Therapeutics and Technology Assessment Subcommittee of the American Academy of Neurology and American Epilepsy Society’, *Neurology* 73(2), 142–149. https://doi.org/10.1212/WNL.0b013e3181a6b325	Should be considered routinely for lamotrigine (seizure frequency is probably increased when 65% of target level is reached). Carbamazepine and phenytoin may be considered.	Not mentioned	Not mentioned
**Epilepsy therapy development project – Epilepsy Foundation of America (ETDP-EFA)** Tomson, T., Battino, D., French, J., Harden, C., Holmes, L., Morrow, J. et al., 2007, ‘Antiepileptic drug exposure and major congenital malformations: The role of pregnancy registries’, *Epilepsy & Behavior* 11(3), 277–282. https://doi.org/10.1016/j.yebeh.2007.08.015	Careful monitoring of AED levels is needed throughout pregnancy. AED levels should be monitored closely in the weeks following delivery as they may increase gradually. Levetiracetam and lamotrigine showed elevated levels within days of delivery in some patients.	Ultrasound at 11–13 weeks is recommended to rule out neural tube defects (along with serum alpha-fetoprotein at 16 weeks) and other major congenital malformations. An ultrasound at 18–22 weeks is also recommended to determine cardiac development, head and spine anatomy and cleft lip or cleft palate.	Recommended visits as required
**United Kingdom – National Institute for Health and Care Excellence (UK NICE)** Gonzalez-Viana, E., Sen, A., Bonnon, A. & Cross, J.H., 2022, ‘Epilepsies in children, young people, and adults: Summary of updated NICE guidance’, *BMJ* 378. https://doi.org/10.1136/bmj.o1446	Recommended if seizures increase or are likely to increase, or if doses need to be adjusted. Not otherwise routinely recommended	Pregnant women and girls who are taking AEDs should be offered a high-resolution ultrasound scan at 18–20 weeks of gestation	Recommended
**Royal College of Obstetricians and Gynaecologists (RCOG) – Green-top guideline no. 68** No, G.T.G., 2016, *Epilepsy in pregnancy*, viewed from https://doctor2017.jumedicine.com/wp-content/uploads/sites/7/2021/07/gtg68_epilepsy.pdf.	There is no clear evidence to show that therapeutic drug monitoring reduces the risk of seizure deterioration compared with monitoring based on clinical features. Clinicians will need to take into account other features such as suspicion of non-adherence, toxicity and intractable seizures in their decisions on therapeutic drug monitoring.	Early pregnancy can be an opportunity to screen for structural abnormalities. The foetal anomaly scan at 18-20 weeks of gestation can identify major cardiac defects in addition to neural tube defects. All WWE should be offered a detailed ultrasound in line with the National Health Service Foetal Anomaly Screening Programme standards.	Recommended when changes to management occur
**South Australian Guideline** Morton, M., Rogers, A., Peat, B. & Hotham, N., 2018, *South Australian Maternal, Neonatal, and Gynaecology Community of Practice,* South Australian Perinatal Practice guideline: Epilepsy and Pregnancy management, ISBN: 9781742430676, viewed from: https://www.sahealth.sa.gov.au/wps/wcm/connect/216779804ee459ccb98cbdd150ce4f37/Epilepsy+and+Pregnancy+Management.	Monitor therapeutic AED levels every 1–2 months to obtain optimal seizure control.	Should occur at 18–20 weeks gestation	Should occur in the first trimester
**Australian Pregnancy Registry** Eadie, M.J., 2020, ‘Antiepileptic drug safety in pregnancy: possible dangers for the pregnant woman and her foetus’, *Evaluation* 15, 13. https://doi.org/10.1007/s40120-021-00252-5	Monitor serum levels of AEDs at least once in each trimester.	Offer ultrasound scans at 11–13 weeks and expert morphology ultrasound scans at 18–20 weeks.	WWE should be seen by obstetricians and neurologists in a multidisciplinary team environment.
**UK Epilepsy and Pregnancy Register** Morrow, J.I., Russell, A., Guthrie, E., Parsons, L., Robertson, I., Waddell, R. et al., 2006, ‘Malformation risks of antiepileptic drugs in pregnancy: A prospective study from the UK Epilepsy and Pregnancy Register’, *Journal of Neurology, Neurosurgery & Psychiatry* 77(2), 193–198. https://doi.org/10.1136/jnnp.2005.074203	Monitor AED levels when doses need to be adjusted, e.g. with phenytoin use.	Recommended at 18–20 weeks of gestation	WWE should be cared for by an obstetrician and physician.
**International Registry of Anti-epileptic Drugs and Pregnancy (EURAP)** EURAP Study Group, 2006, ‘Seizure control and treatment in pregnancy: Observations from the EURAP epilepsy pregnancy registry’, *Neurology* 66(3), 354–360. https://doi.org/10.1212/01.wnl.0000195888.51845.80	Not mentioned	Not mentioned	Not mentioned

AED, anti-epileptic drug; WWE, women with epilepsy.

### South African guidelines

#### Supplementation and medicine recommendations

The National Department of Health (NDoH) epilepsy guidelines recommend that AEDs should only be commenced in patients at high risk of subsequent seizures following a first unprovoked seizure (e.g. abnormal neurological examination, strong family history, abnormal brain imaging). Advanced counselling is recommended so that patients are aware of the adverse effects of alcohol on seizures, the effect of missing a dose of medication and the importance of sleep hygiene (NDoH 2020). Also, women of child-bearing age should be informed about the teratogenic effects of AEDs (NDoH 2020). The guideline also makes note of the possible drug–drug interactions between AEDs and hormonal contraceptives (Table 1). Treatment aims to achieve optimal seizure control using monotherapy (NDoH 2020). Monotherapy has been encouraged as the ideal in epilepsy treatment due to reduced side effects, prevention of drug–drug interactions, improved patient compliance, lower cost and, in many cases, improve seizure control compared to polytherapy (Table 1). Patients with a history of myoclonic seizures or typical absence seizures should preferably be treated with sodium valproate as those seizures may be aggravated by the use of either phenytoin or carbamazepine (NDoH 2020). The goal of treatment in pregnancy is to achieve optimal seizure control using monotherapy, while avoiding sodium valproate use (NDoH 2020). Folic acid supplementation is also recommended prior to falling pregnant at a dose of 5 mg daily, orally (NDoH 2020).

#### Monitoring during pregnancy

Pregnancy alters AED serum concentration levels, and, therefore, AED doses should be adjusted accordingly (NDoH 2020). Current epilepsy guidelines in SA advise on commencing management by obtaining a patient history and conducting a computed tomography (CT) scan, followed by other investigations that may be clinically warranted (NDoH 2020). Patients are advised to record dates and times of seizures in a seizure diary and present this at each consultation for assessment of therapy (Table 1). The guidelines also stipulate that routine therapeutic drug monitoring is not useful except under certain circumstances, which may be to confirm toxicity in a symptomatic patient or identify poor patient adherence (Table 1).

### International guidelines

#### Risks, anti-epileptic drugs and seizure-induced harm during pregnancy

Seizure-induced harm during pregnancy can potentially increase the risk of injuries and/or hypoxia in the foetus as outlined by numerous guidelines mentioned in Table 2. The United Kingdom – National Institute for Health and Care Excellence (UK NICE) guideline has suggested that tonic-clonic seizures in particular have a relatively higher risk of harm to the foetus (Table 2). In addition, some of these guidelines have advised that seizure frequency during pregnancy is more likely to increase in 15% – 37% of WWE (Table 2). The North American Registry and The Epilepsy Therapy Development Project – Epilepsy Foundation of America (ETDP-EFA) have advised that there could be an increased risk of vaginal bleeding, hypertension, preeclampsia, antepartum haemorrhage and caesarean delivery in pregnant WWE (Table 2). In addition, neonates have an increased risk of low birth weight (Table 2). The UK Epilepsy and Pregnancy Register as well as several other guidelines have advised there could be a 2/3-fold increase in miscarriage and intrauterine growth restriction in the foetus (Table 2). With reference to AED-induced harm to the foetus, multiple guidelines have highlighted the possible life-threatening risks imposed onto the foetus when AEDs (e.g. sodium valproate) that are not recommended are taken (Table 2). The Royal College of Obstetrics and Gynaecologists (RCOG – Green-top) guideline shows an increased risk of major congenital malformations in children exposed to AEDs in utero when compared with the general population (Table 2). The exposure to AEDs in utero is associated with adverse development (gross motor skills, sentence skills, autistic traits), minor abnormalities, microcephaly as well as lower IQ scores in the newborn (Table 2).

#### Supplementation and medicine recommendations

The American Academy of Neurology (AAN) and American Epilepsy Society (AES) have presented the benefits of administering preconception folic acid and vitamin K supplementation at birth in their guidelines (Table 3). Folate deficiencies have been associated with neural tube defects in the general population and supplementation with folic acid (400 mcg – 500 mcg) has been shown to reduce risks of neural tube defects by 60% – 86% in the general population (Table 3). The ETDP-EFA recommends a dose of 600 mcg for pregnant WWE and a dose of 500 mcg for lactating women (Table 3).

According to the South Australian Perinatal Practice guideline, antenatal vitamin K is administered to women taking hepatic enzyme-inducing AEDs to avoid bleeding in the newborn (Table 3). The ETDP-EFA endorses an oral dose of 10 mg/day – 20 mg/day of vitamin K during the last month of pregnancy for women taking enzyme-inducing AEDs and 1 mg to be administered parentally (Intravenous or Intramuscular) to the baby at birth (Table 3).

The International Registry of Anti-epileptic Drugs and Pregnancy as well as several other guidelines have warned against the use of sodium valproate, carbamazepine, phenobarbitone and phenytoin in pregnant WWE (Table 3). In addition, many guidelines have therefore recommended the use of other AEDs such as lamotrigine and levetiracetam (Table 3).

Polytherapy during the first trimester is associated with an increased risk of major congenital malformations in the newborn. (Table 3). The AAN and AES have advised that AED polytherapy should be avoided in pregnancy to decrease the risk of major congenital malformations and therefore monotherapy is preferred while still maintaining seizure control (Table 3).

#### Monitoring during pregnancy

The UK NICE guidelines endorse high-resolution ultrasounds only at 18–20 weeks of gestation, while the ETDP-EFA recommends the above at 11–13 and 18–22 weeks of gestation to determine the presence of major congenital malformations, cardiac development, head and spine anatomy and cleft lip/palate. According to the RCOG – Green-top guidelines, all WWE should be offered a detailed ultrasound in line with the National Health Service Foetal Anomaly Screening Programme standards (Table 4). The above-mentioned guidelines also encourage routine serum AED level monitoring for the following reasons; to establish the lowest therapeutic dose, if seizure frequency increases or is likely to increase, or if dose adjustments are required. The South Australian Guideline and numerous other guidelines have mentioned that therapeutic drug monitoring should be regularly conducted in order to achieve optimal seizure control and prevent drug toxicities (Table 4). In addition, the lowest therapeutic dose for each AED is advised for use during pregnancy (Table 4). These guidelines endorse that pregnant WWE should be regularly overseen by a team of obstetricians and neurologists (Table 4).

## Discussion

Prescribing AEDs in pregnancy remains a challenge to many clinicians. A host of questions arise that must be addressed even before conception. In WWE, it may be unsafe to stop or even change an AED regimen as these changes could be injurious to health and lead to fatalities in both mother and child (Gedzelman & Meador 2012). Many international studies, together with the South African epilepsy guidelines, have recommended that the use of AED monotherapy is preferred before and during pregnancy as it can reduce the risk of long-term poor cognitive outcomes (Harris & Hope 2023; NDoH 2020). According to a recent study, the risk of malformations was 3.1% – 7.8% in infants exposed to AED monotherapy, 8.3% – 13.5% in infants exposed to two AEDs, and more than 13.5% in infants exposed to three AEDs (Gedzelman & Meador 2012; Harris & Hope 2023).

Several international guidelines have explained the use of antenatal vitamin K (10 mg per day – 20 mg per day in the last month of pregnancy) and that it should be administered to WWE taking hepatic enzyme-inducing AEDs to avoid bleeding in the newborn (South Australian Perinatal Practice Guideline 2018); however, no mention of antenatal vitamin K usage is made in the South African guidelines (NDoH 2020). In numerous practices in the US, UK and Australia, WWE planning a pregnancy are recommended 400 mcg of folic acid a day (Eadie 2020; Epilepsy org 2022). Folate deficiencies have been associated with neural tube defects in the general population (Wald 2022). A study conducted in the US confirmed that preconception use of folic acid (500 mcg per day) in WWE resulted in no major congenital malformations, whereas no folic acid use was associated with 23% increase in foetal abnormalities (Wald 2022).

As seizures carry the risk of potential injury or even death, most WWE cannot afford to discontinue AEDs (Harris & Hope 2023). It is therefore essential to define which AEDs produce optimal seizure control during pregnancy while minimising teratogenicity (Borgelt et al. 2019; Harden et al. 2009). A key part of preparing a WWE for pregnancy involves identifying the minimum therapeutic dose that can control their seizures (Wald 2022). However, despite monotherapy offering notably lower risks during pregnancy, each AED possesses its own associated teratogenic risk when used as monotherapy and should therefore be considered on an individual patient basis (Arfman et al. 2020).

The cause for any increase in seizures during pregnancy is not clearly understood and is likely to be multifactorial (Hussein, Kai & Qureshi 2016). Pregnancy is associated with several physiological, endocrine and psychological changes, any or all of which might contribute to lowering the seizure threshold (Hussein et al. 2016). Physiological changes during pregnancy alter the pharmacokinetics of AEDs, which may result in lower therapeutic drug levels and seizure deterioration in WWE (Hussein et al. 2016; Patel & Pennell et al. 2022). Once a WWE has conceived, appropriate evaluation and counselling should continue along with frequent risk–benefit pharmacotherapy evaluations and monthly monitoring of AED serum concentration levels; however, current epilepsy guidelines used in SA do not recommend therapeutic drug monitoring unless the patient exhibits signs of AED toxicities, which will then require levels to be monitored (Khan et al. 2020; NDoH 2020).

Studies conducted in the US indicate that it is common practice to recommend a level II ultrasound at 18–20 weeks of gestation (Pennell et al. 2022). This is a detailed anatomic evaluation, which provides very high sensitivity for structural abnormalities affecting the foetus. However, guidelines used in SA advise a CT scan at the commencement of treatment only (NDoH 2020). No further information regarding the use of high-resolution ultrasounds have been made in these guidelines (NDoH 2020).

Because of a high risk of teratogenicity and cognitive impairment with sodium valproate and phenobarbitone, nearly all guidelines (SA included) have suggested that these two AEDs should never be initiated in a pregnant patient or women of childbearing age (Wald 2022). Many other studies have suggested that carbamazepine and phenytoin should also be avoided because of the increased risk of cleft palate, cardiac malformations and poor cognitive outcomes (Harris & Hope 2023; Schmidt & Schachter 2014). Lamotrigine currently has the most data available that indicates no substantial risk of major congenital malformations; in addition, preliminary studies have found a good safety profile for levetiracetam in pregnancy (Arfman et al. 2020; Pennell et al. 2022).

Therefore, the presence of epilepsy and exposure to AEDs presents a significant association with adverse outcomes in pregnancy. The increased risks and data regarding each AED should be considered when discussing reproductive health and conception planning for WWE when developing policies and guidelines to manage the condition (Borgelt et al. 2019). As stipulated in the NDoH guidelines, oral contraceptives and the progestin subdermal implant are unreliable contraceptive options for WWE because of the enzyme-inducing activity of AEDs. The risk of oral contraceptive failure, which increases seven-fold when taken with AEDs, are well documented in the South African epilepsy guidelines but was scarcely mentioned in any international guide. An evidence-based approach, using recommendations from leading organisations needs to be reviewed and incorporated into policies developed for managing patients with epilepsy before, during and after pregnancy (Patel & Pennell 2016).

### Limitations

- It is possible that not all guidelines have been published online
- Some guidelines may have limited or restricted access (e.g. those that are made available to specific countries only)
- Guidelines may not have been updated recently

## Conclusion

The clinical management of WWE on AEDs during pregnancy is perplexing. The accurate management of pregnant WWE should include a vast array of stepwise protocols such as patient education, frequent monitoring of AED serum levels, thorough patient counselling, potential preconception changing of antiepileptic medications, applying AED dosage adjustments, minimising toxic drug concentrations, as well as avoiding potentially teratogenic medications (Borgelt et al. 2019). Folic acid supplementation is also recommended before falling pregnant at a dose of 500 mcg daily, orally, as well as vitamin K supplementation at birth (Eadie 2020; South Australian Perinatal Practice Guideline 2018).

Physiological changes during pregnancy alter the pharmacokinetics of AEDs, which may result in lower serum AED levels and seizure deterioration. Patients should be carefully educated on potential major congenital malformations, neurodevelopmental outcomes, obstetrical risks, perinatal complications and breastfeeding while on AEDs. It is essential to monitor WWE during pregnancy and despite multiple complexities involving their care, it is imperative to manage these patients efficiently to ensure positive outcomes in pregnancy (Arfman et al. 2020; Pennell et al. 2023). An individualised approach delivered by a team of neurologists, obstetricians, primary care doctors, nurses and clinical pharmacists with knowledge of various aspects of epilepsy in pregnancy is needed to improve outcomes in pregnant WWE.

Currently, in addition to existing epilepsy guidelines for the general population, there are no protocols and prescribing guidelines, other than a step-wise medicine treatment specific to the management of pregnant WWE in SA (NDoH 2020). There have not been any published studies conducted in SA to assess the compliance to NDoH antiepileptic guidelines for pregnant women and women of child-bearing age. Furthermore, these guidelines have not been compared to the management strategies prescribed by the WHO or other developed countries. It is therefore imperative to assess whether the prescribed guidelines and precautions are being used to treat these patients and which of these steps can be improved.
